# Prevalence of Cefixime-Resistant *Neisseria gonorrhoeae* in Melbourne, Australia, 2021–2022

**DOI:** 10.1093/infdis/jiae313

**Published:** 2024-06-15

**Authors:** Eric P F Chow, Kerrie Stevens, Vesna De Petra, Marcus Y Chen, Catriona S Bradshaw, Norelle L Sherry, Lindley A Barbee, Lenka A Vodstrcil, Ivette Aguirre, Kate L Seib, Kate Maddaford, Deborah A Williamson, Benjamin P Howden, Christopher K Fairley

**Affiliations:** Melbourne Sexual Health Centre, Alfred Health, Melbourne, Victoria, Australia; School of Translational Medicine, Faculty of Medicine, Nursing and Health Sciences, Monash University, Melbourne, Victoria, Australia; Centre for Epidemiology and Biostatistics, Melbourne School of Population and Global Health, The University of Melbourne, Melbourne, Victoria, Australia; Microbiological Diagnostic Unit Public Health Laboratory, Department of Microbiology and Immunology, University of Melbourne, at the Peter Doherty Institute for Infection and Immunity, Melbourne, Victoria, Australia; Melbourne Sexual Health Centre, Alfred Health, Melbourne, Victoria, Australia; Microbiological Diagnostic Unit Public Health Laboratory, Department of Microbiology and Immunology, University of Melbourne, at the Peter Doherty Institute for Infection and Immunity, Melbourne, Victoria, Australia; Melbourne Sexual Health Centre, Alfred Health, Melbourne, Victoria, Australia; School of Translational Medicine, Faculty of Medicine, Nursing and Health Sciences, Monash University, Melbourne, Victoria, Australia; Melbourne Sexual Health Centre, Alfred Health, Melbourne, Victoria, Australia; School of Translational Medicine, Faculty of Medicine, Nursing and Health Sciences, Monash University, Melbourne, Victoria, Australia; Centre for Epidemiology and Biostatistics, Melbourne School of Population and Global Health, The University of Melbourne, Melbourne, Victoria, Australia; Microbiological Diagnostic Unit Public Health Laboratory, Department of Microbiology and Immunology, University of Melbourne, at the Peter Doherty Institute for Infection and Immunity, Melbourne, Victoria, Australia; Department of Infectious Diseases and Immunology, Austin Health, Heidelberg, Victoria, Australia; Department of Medicine, University of Washington, Seattle, Washington, USA; Public Health–Seattle and King County HIV/STD Program, Seattle, Washington, USA; Melbourne Sexual Health Centre, Alfred Health, Melbourne, Victoria, Australia; School of Translational Medicine, Faculty of Medicine, Nursing and Health Sciences, Monash University, Melbourne, Victoria, Australia; Centre for Epidemiology and Biostatistics, Melbourne School of Population and Global Health, The University of Melbourne, Melbourne, Victoria, Australia; Melbourne Sexual Health Centre, Alfred Health, Melbourne, Victoria, Australia; Institute for Glycomics, Griffith University, Southport, Queensland, Australia; Melbourne Sexual Health Centre, Alfred Health, Melbourne, Victoria, Australia; School of Translational Medicine, Faculty of Medicine, Nursing and Health Sciences, Monash University, Melbourne, Victoria, Australia; Victorian Infectious Disease Reference Laboratory, The Royal Melbourne Hospital, at the Peter Doherty Institute for Infection and Immunity, Melbourne, Victoria, Australia; Department of Infectious Diseases, University of Melbourne, at the Peter Doherty Institute for Infection and Immunity, Melbourne, Victoria, Australia; Department of Infectious Diseases and Immune Defence, Walter and Eliza Hall Institute of Medical Research, Melbourne, Victoria, Australia; Microbiological Diagnostic Unit Public Health Laboratory, Department of Microbiology and Immunology, University of Melbourne, at the Peter Doherty Institute for Infection and Immunity, Melbourne, Victoria, Australia; Department of Infectious Diseases and Immunology, Austin Health, Heidelberg, Victoria, Australia; Centre for Pathogen Genomics, The University of Melbourne, Melbourne, Victoria, Australia; Melbourne Sexual Health Centre, Alfred Health, Melbourne, Victoria, Australia; School of Translational Medicine, Faculty of Medicine, Nursing and Health Sciences, Monash University, Melbourne, Victoria, Australia

**Keywords:** antimicrobial resistance, gonorrhea, *Neisseria gonorrhoeae*, resistance, treatment

## Abstract

While ceftriaxone remains the first-line treatment for gonorrhea, the US Centers for Disease Control and Prevention recommended cefixime as a second-line treatment in 2021. We tested 1176 *Neisseria gonorrhoeae* isolates among clients attending the Melbourne Sexual Health Centre in 2021 and 2022. The prevalence of cefixime resistance was 6.3% (74/1176), azithromycin resistance was 4.9% (58/1176), and ceftriaxone resistance was 0% (0/1176). Cefixime resistance was highest among women (16.4%, 10/61), followed by men who have sex with women (6.4%, 7/109) and men who have sex with men (5.8%, 57/982). The prevalence of cefixime-resistant *N gonorrhoeae* exceeds the threshold of the 5% resistance level recommended by the World Health Organization; thus, cefixime treatment would have limited benefits in Australia.

## Teaser

The prevalence of cefixime-resistant *Neisseria gonorrhoeae* among sexually active individuals in Melbourne exceeds the threshold of the 5% resistance level recommended by the World Health Organization. Thus, cefixime treatment would have limited benefits in Australia.

Sexually transmitted infections (STIs) have been rising worldwide over the last decade [1]. Gonorrhea is of particular concern because cases resistant to ceftriaxone, the last remaining fully effective antimicrobial against *Neisseria gonorrhoeae*, have occurred [2]. Dual antimicrobial therapy (ceftriaxone plus azithromycin) was introduced in the early 2010s for first-line treatment for gonorrhea in many countries. However, several countries, including the United States [3] and United Kingdom [4], changed to ceftriaxone monotherapy in the late 2010s due to observed rises in azithromycin resistance in *N gonorrhoeae* after the introduction of dual therapy and the likelihood of selecting for macrolide-resistant *Mycoplasma genitalium* [5]. Alternative regimens for gonorrhea treatment are urgently required to address the rise in antimicrobial resistance in *N gonorrhoeae*.

Cefixime is a third-generation oral cephalosporin antibiotic. In 2002, the US Centers for Disease Control and Prevention recommended a single oral dose of cefixime (400 mg) as one of the first-line treatments for gonorrhea [6]. In 2012, this recommendation ceased because the rising rates of cefixime resistance raised concerns about the increasing resistance in all cephalosporins. In 2021, the Centers for Disease Control and Prevention recommended a single dose of ceftriaxone (500 mg) as the only first-line treatment for uncomplicated gonorrhea; however, a single oral dose of cefixime (800 mg) can be considered an alternative treatment if ceftriaxone administration is not available or feasible [3]. Cefixime was not considered the first-line treatment because of decreased susceptibility or resistance in surveillance data and the concerns about its effectiveness for oropharyngeal gonorrhea treatment [7].

A global antimicrobial resistance surveillance study for *N gonorrhoeae* in 2017 to 2018 found that only 51 countries reported data on cefixime susceptibility, and there have been no data in Australia [8]. Thus, we conducted this study aimed to determine the prevalence of cefixime-resistant *N gonorrhoeae* among individuals attending a sexual health clinic in Melbourne, Australia.

## METHODS

This cross-sectional study was conducted at the Melbourne Sexual Health Centre (MSHC) between 9 August 2021 and 18 July 2022. The MSHC is a major public sexual health service in Victoria, Australia. It provides about 50 000 clinical consultations each year. Individuals at higher risk for STIs and those with STI symptoms were preferentially triaged into the clinic. HIV and STI testing and treatment are free of charge.

Individuals were screened for *N gonorrhoeae* by nucleic acid amplification test with the Aptima Combo 2 Assay (Hologic Panther system; Hologic). Untreated individuals with a positive test result were recalled to the MSHC for treatment, and a swab was taken for culture. Swabs were inoculated onto the Modified Thayer Martin Agar with a selective medium that contains a GC agar base (Difco; supplied by the Media Preparation Unit, The University of Melbourne) [9] and incubated at 35 to 37 °C in 5% CO_2_ for 48 hours. Oxidase-positive gram-negative diplococci colonies resembling *N gonorrhoeae* underwent confirmatory testing by the Vitek-MS MALDI TOF system (BioMérieux). Ceftriaxone, cefixime, and azithromycin minimum inhibitory concentrations (MICs) were determined by agar dilution at the Microbiological Diagnostic Unit Public Health Laboratory in Victoria, Australia. MIC testing was performed from a 20- to 24-hour culture on chocolate agar incubated at 35 ± 1 °C in 5% CO_2_. In some cases, isolates were stored in glycerol storage broth at −80 °C prior to subculture. MIC plates were prepared per standard M07 of the Clinical and Laboratory Standards Institute via antimicrobial powders obtained from Sigma Aldrich (ceftriaxone, ciprofloxacin, penicillin, spectinomycin, and tetracycline) or Pfizer (azithromycin) [10]. The European Committee on Antimicrobial Susceptibility Testing (EUCAST) breakpoint tables and interpretive criteria were used to interpret the MICs [11]. Isolates with ceftriaxone or cefixime MICs >0.125 mg/L were considered resistant and azithromycin MICs >1 mg/L as likely resistant (EUCAST epidemiologic cutoff value).

For individuals with isolates from multisite infections collected on the same day (eg, genital, oropharyngeal, and rectal), we randomly selected 1 isolate per individual and excluded the remaining isolates. For those with samples collected on different days during the study period, the isolates from different episodes (1 per episode) were included in the final analysis. In other words, only 1 isolate per patient episode was included in the final analysis.

We calculated the proportion of isolates with resistance to cefixime, azithromycin, and ceftriaxone and the corresponding 95% CIs with the exact binomial method. Univariable logistic regression with a generalized estimating equation was performed to identify factors that were associated with cefixime-resistant *N gonorrhoeae*. Variables with a *P* value <.20 in the univariable analyses were included in the multivariable analyses. The exchangeable correlation matrix was used. Crude and adjusted odds ratios and the corresponding 95% CIs were reported. All statistical analyses were performed in Stata (version 17; StataCorp). This study was approved by the Alfred Hospital Ethics Committee, Melbourne, Australia (685/22).

## RESULTS

Between August 2021 and July 2022, 1297 *N gonorrhoeae* isolates from 1049 individuals underwent antimicrobial susceptibility testing for cefixime, azithromycin, and ceftriaxone​. Of the 114 patients who had more than 1 isolate, we randomly selected 1 isolate per person per episode, and the remaining 121 isolates were excluded. The remaining 1176 isolates from 1049 clients were included in the final analysis. Of the 1176 isolates, 104 people had isolates from multiple episodes (126 isolates) and the median time between episodes per person was 95 days (IQR, 59–139).

The median age of the individuals was 31 years (IQR, 27–37), and most were gay, bisexual, and other men who have sex with men (MSM; 83.5%, 982/1176). About 71.5% (841/1176) were HIV negative and not taking HIV preexposure prophylaxis (PrEP); 17.0% (200/1176) were taking HIV PrEP; and 11.5% (135/1176) were people with HIV. Most isolates were collected from urogenital sites (including urethral, vaginal, and cervical specimens; 40.2%, 473/1176), followed by the rectum (35.8%, 421/1176) and oropharynx (24.0%, 282/1176).

**Figure 1.**
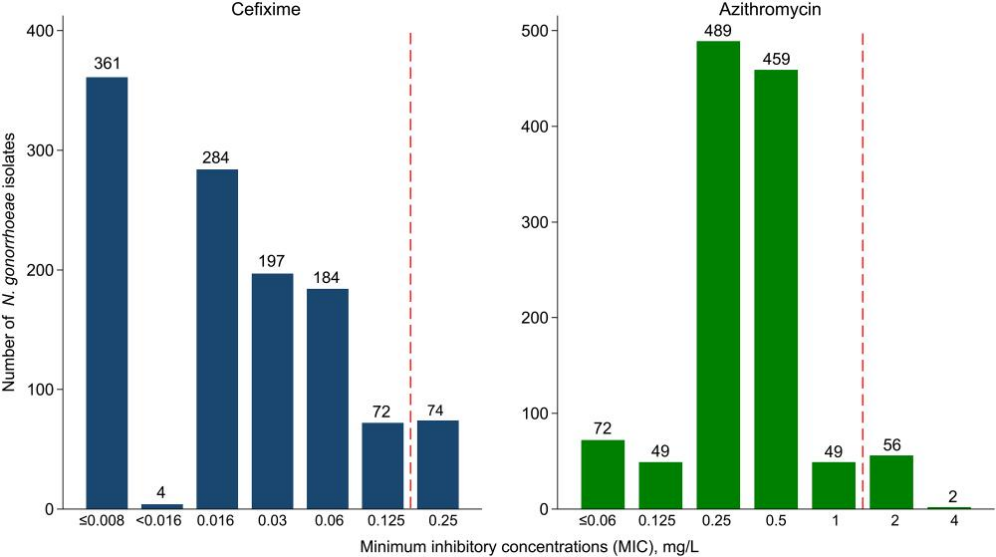
The distribution of minimum inhibitory concentrations of cefixime and azithromycin for *Neisseria gonorrhoeae* isolates. The vertical line indicates the resistance breakpoint.

The proportion of isolates with resistance to cefixime was 6.3% (95% CI, 5.0%–7.8%; 74/1176), and azithromycin was 4.9% (95% CI, 3.8%–6.3%; 58/1176; Figure 1). There were no isolates with resistance to cefixime and azithromycin. No isolates demonstrated resistance to ceftriaxone (0%; 95% CI, 0%–.3%; 0/1176). Cefixime resistance rates varied by anatomic site, with 7.4% (21/282) resistance at the oropharynx, followed by 6.3% (30/473) from urogenital sites and 5.5% (23/421) from the rectum. Women had the highest level of cefixime resistance (16.4%, 10/61), followed by men who have sex with women (6.4%, 7/109) and MSM (5.8%, 57/982).

Multivariable logistic regression showed that women had higher odds (adjusted odds ratio, 2.72; 95% CI, 1.22–6.06) of being infected with *N gonorrhoeae* resistant to cefixime as compared with MSM after adjusting for continent of birth and sex work status (Table 1). Cefixime resistance was not associated with age, anatomic site, HIV status, PrEP use, and sex overseas in the last 12 months. Of the 10 women carrying cefixime-resistant isolates of *N gonorrhoeae*, most of them were born in Asia (n = 6); half of them had worked as sex workers (n = 5); and the isolates were collected from the vagina (n = 5), followed by the cervix (n = 2), oropharynx (n = 2), and urine (n = 1).

**Table 1.** Characteristics Associated With Cefixime-Resistant *Neisseria gonorrhoeae* Among 1176 Isolates From 1049 Individuals

Characteristics	No. (%)	OR (95% CI)	*P* Value	Adjusted OR (95% CI)	*P* Value
Age	…	1.01 (.98–1.03)	.652	…	…
Specimen type					
Urogenital^a^	30/473 (6.3)	1 [Reference]		…	…
Oropharyngeal	21/282 (7.4)	1.18 (.66–2.12)	.567		
Rectal	23/421 (5.5)	0.85 (.49–1.50)	.581		
Population					
MSM^b^	57/982 (5.8)	1 [Reference]		1 [Reference]	
MSW^c^	7/109 (6.4)	1.12 (.49–2.52)	.791	1.20 (.53–2.76)	.660
Women	10/61 (16.4)	3.16 (1.51–6.59)	.002	2.72 (1.22–6.06)	.014
Transgender or other gender	0/24 (0)	…	…	…	…
HIV status and PrEP use					
People living with HIV	5/135 (3.7)	1 [Reference]		…	…
HIV negative	53/841 (6.3)	1.75 (.67–4.55)	.252		
PrEP users	16/200 (8.0)	2.24 (.78–6.40)	.132		
Continent of birth					
Oceania	35/655 (5.3)	1 [Reference]		1 [Reference]	
Europe	7/93 (7.5)	1.45 (.62–3.39)	.387	1.46 (.62–3.42)	.387
Asia	24/288 (8.3)	1.62 (.94–2.79)	.085	1.68 (.97–2.91)	.064
Africa	2/24 (8.3)	1.58 (.34–7.35)	.560	1.70 (.37–7.7)	.492
North America	0/20 (0)	…	…	…	…
South America	5/77 (6.5)	1.25 (.47–3.35)	.654	1.34 (.50–3.59)	.558
Unknown	1/19 (5.3)	1.00 (.13–7.70)	.999	1.18 (.15–9.17)	.874
Sex work					
Never	66/1105 (6.0)	1 [Reference]		1 [Reference]	
Ever	8/71 (11.3)	1.97 (.89–4.37)	.093	1.71 (.71–4.13)	.230
Sex overseas in the last 12 mo					
No	27/423 (6.4)	1 [Reference]		…	…
Yes	2/56 (3.6)	0.55 (.13–2.37)	.427		
Unknown	45/697 (6.5)	1.01 (.62–1.65)	.969		

Abbreviations: OR, odds ratio; PrEP, preexposure prophylaxis.

^a^Urogenital sites include urethral, vaginal, and cervical specimens.

^b^Men who have sex with men and women (last 12 months).

^c^Men who have sex with women only (last 12 months).

## DISCUSSION

The proportion of isolates with resistance to cefixime was 6.3% among sexually active individuals attending an Australian sexual health clinic. This is above the maximum 5% antimicrobial resistance rate recommended by the World Health Organization (WHO) for an antimicrobial to be suitable as a standard treatment regime. Our previous study tested 7588 *N gonorrhoeae* isolates in Victoria and found that the prevalence of resistance to ceftriaxone was 2.9% and that to azithromycin was 8.2% in 2018 [12]. This level of azithromycin resistance (>5% per the WHO-recommended antimicrobial resistance threshold) among isolates of *N gonorrhoeae* from Victoria is of concern as it threatens the efficacy of dual antimicrobial therapy (ceftriaxone plus azithromycin), which is currently recommended as first-line treatment for gonorrhea in Australia's guidelines [13]. With the concern of antimicrobial resistance, the MSHC dropped azithromycin for gonorrhea treatment in late 2021. Furthermore, ceftriaxone plus doxycycline instead of azithromycin is recommended for rectal gonorrhea and chlamydia coinfections per the Australian STI guidelines [14]. Other strategies will be required to counteract the rise in antimicrobial resistance in *N gonorrhoeae*.

Our data showed that 6.3% of *N gonorrhoeae* isolates among clinic attendees were resistant to cefixime. This estimate is similar to estimates from some European countries, such as Italy (5.0%, 5/100), Switzerland (5.6%, 3/54), and Greece (6.0%, 5/83), but substantially higher than that in the United States (0.3%, 15/5160) [8]. However, the United States uses the Clinical and Laboratory Standards Institute susceptibility breakpoint for cefixime (MIC ≤0.25 mg/L) [15], which is higher than the EUCAST's breakpoint (MIC <0.125 mg/L) [11]. We also found that women (n = 61) have a higher level of cefixime resistance (16%) when compared with men (6%). We found that most women with isolates resistant to cefixime were born in Asia. It is possible that these women may have been exposed to cefixime in the past for other infections, including urinary tract infections, or they might have had sex with individuals from Asia or within a sexual network where cefixime-resistant *N gonorrhoeae* might have been imported from Asia. There have been limited data on the prevalence of resistance to cefixime in Asia [8]. In 2022, a Chinese study tested 2804 *N gonorrhoeae* isolates and reported a high prevalence of resistance to cefixime (16.0%) with a MIC >0.125 mg/L as the breakpoint [16]. Further genomic work may be required to identify the *N gonorrhoeae* strain with resistance to cefixime to better understand why a high level of resistance is concentrated in certain populations.

The major strengths of this study are its large sample size (N = 1176) and that it represents the first data on cefixime susceptibility from Australia. The 2017–2018 WHO global antimicrobial resistance surveillance data showed that of the 51 countries that reported data on cefixime susceptibility, the Philippines was the only country in the Western Pacific region to do so (4.2%, 4/95), and just 3 countries (ie, United States, Canada, and Chile) tested >1000 isolates [8]. Our study provides additional estimates not only in Australia but also in the Western Pacific region. However, our study has several limitations. First, it was conducted at a single urban sexual health service, and sexual health clinic attendees may have had higher levels of antibiotic exposure or more diverse sexual networks as compared with the general population. Additionally, about half the attendees at our clinics are travelers where importation of resistant *N gonorrhoeae* may have occurred, and this may not fully represent the prevalence of resistant *N gonorrhoeae* in the broader population [17, 18]. Most data in other countries, though, come from STI centers and, like our study, may have overestimated the prevalence of resistance in the entire community. Second, this study was conducted in 2021 to 2022 with major lockdowns during the COVID-19 pandemic. Clinic attendees during this period might have different characteristics due to the changes in sexual practices and STI screening patterns during COVID-19 [19]. The lockdowns also meant that there was likely to be less international travel; as such, our data are more likely than usual to represent locally endemic strains rather than recently imported ones.

Cefixime is currently not available in Australia. As the current prevalence of cefixime-resistant *N gonorrhoeae* among sexually active individuals in Melbourne exceeds the WHO's recommended antimicrobial resistance threshold of 5%, these data suggest that cefixime would have limited benefits for gonorrhea treatment in Australia. Additionally, while a meta-analysis concluded that cefixime (400 and 800 mg) for gonorrhea treatment is highly effective in treating urogenital infections (97% and 98% cure rates, respectively), it is less effective for eradicating oropharyngeal infections (91% and 82% cure rates) [7]. There are limited options available for first-line treatment of gonorrhea; thus, further development and investigation of alternative therapies for gonorrhea treatment are required.

## Notes

***Acknowledgment*.** We thank Rohit Sasidharan at the Melbourne Sexual Health Centre for his help in extracting electronic data for the study and the technical staff at the Microbiological Diagnostic Unit Public Health Laboratory for performing antimicrobial susceptibility testing.

***Author contributions.*** E. P. F. C., L. A. B., D. A. W., and C. K. F. conceived the study. E. P. F. C. performed data analyses, interpreted data, and wrote the manuscript. K. S., V. D. P., D. A. W., and B. P. H. supervised the laboratory testing. D. A. W. and B. P. H. provided laboratory support on antimicrobial susceptibility testing. K. S., V. D. P., N. L. S., K. L. S., D. A. W., and B. P. H. helped with data interpretation on antimicrobial susceptibility testing. M. Y. C., C. S. B., L. A. B., L. A. V., I. A., K. M., and C. K. F. helped with data interpretation on clinical perspective and antimicrobial resistance. All authors revised the manuscript for intellectual content and approved the final version of the manuscript. The corresponding author had full access to all the data in the study and had final responsibility for the decision to submit for publication.

***Data availability.*** All data generated or analyzed during this study are included in this published article.

***Financial support*.** This work was supported by an National Health and Medical Research Council Emerging Leadership Investigator Grant (GNT1172873 to E. P. F. C. and GNT1174555 to D. A. W.); and an National Health and Medical Research Council Leadership Investigator Grant (GNT1172900 to C. K. F., GNT1173361 to C. S. B., GNT2017383 to K. L. S., and GNT1196103 to B. P. H.).

## References

[1] Kirkcaldy RD , Weston E, Segurado AC, Hughes G. Epidemiology of gonorrhoea: a global perspective. Sex Health 2019; 16:401–11. 31505159 10.1071/SH19061 PMC7064409

[2] Pleininger S , Indra A, Golparian D, et al Extensively drug-resistant (XDR) *Neisseria gonorrhoeae* causing possible gonorrhoea treatment failure with ceftriaxone plus azithromycin in Austria, April 2022. Euro Surveill 2022; 27(24):2200455. 35713023 10.2807/1560-7917.ES.2022.27.24.2200455 PMC9205165

[3] Workowski KA , Bachmann LH, Chan PA, et al Sexually transmitted infections treatment guidelines, 2021. MMWR Recomm Rep 2021; 70:1–187. 10.15585/mmwr.rr7004a1 PMC8344968 34292926

[4] Fifer H , Saunders J, Soni S, Sadiq ST, FitzGerald M. 2018 UK national guideline for the management of infection with *Neisseria gonorrhoeae*. Int J STD AIDS 2020; 31:4–15. 31870237 10.1177/0956462419886775

[5] Unemo M , Workowski K. Dual antimicrobial therapy for gonorrhoea: what is the role of azithromycin? Lancet Infect Dis 2018; 18:486–8. 29523498 10.1016/S1473-3099(18)30162-2 PMC6748319

[6] Centers for Disease Control and Prevention . Sexually transmitted diseases treatment guidelines 2002. MMWR Recomm Rep 2002; 51:1–78. 12184549

[7] Yang KJ , Kojima N, Bristow CC, Klausner JD. Effectiveness of cefixime for the treatment of *Neisseria gonorrhoeae* infection at 3 anatomic sites: a systematic review and meta-analysis. Sex Transm Dis 2023; 50:131–7. 36729626 10.1097/OLQ.0000000000001742 PMC9906985

[8] Unemo M , Lahra MM, Escher M, et al WHO global antimicrobial resistance surveillance for *Neisseria gonorrhoeae* 2017–18: a retrospective observational study. Lancet Microbe 2021; 2:e627–36. 35544082 10.1016/S2666-5247(21)00171-3

[9] Martin JE , Armstrong JH, Smith PB. New system for cultivation of *Neisseria gonorrhoeae*. Appl Microbiol 1974; 27:802–5. 4207764 10.1128/am.27.4.802-805.1974 PMC380138

[10] Clinical and Laboratory Standards Institute . Methods for dilution antimicrobial susceptibility tests for bacteria that grow aerobically. 11th ed. CLSI standard M07. Wayne, PA: Clinical and Laboratory Standards Institute, 2018.

[11] European Committee on Antimicrobial Susceptibility Testing . Breakpoint tables for interpretation of MICs and zone diameters. Version 14.0. **2024**. https://www.eucast.org/fileadmin/src/media/PDFs/EUCAST_files/Breakpoint_tables/v_14.0_Breakpoint_Tables.pdf. Accessed 17 January 2024.

[12] Williamson DA , Fairley CK, Howden BP, et al Trends and risk factors for antimicrobial-resistant *Neisseria gonorrhoeae*, Melbourne, Australia, 2007 to 2018. Antimicrob Agents Chemother 2019; 63:e01221-19. 31383663 10.1128/AAC.01221-19 PMC6761556

[13] Bourne C , Chen M, Lahra M, et al Recommendations for treatment of gonococcal infections in the era of MDR/XDR gonorrhoea (document for sexual health and infectious diseases specialists). https://ashm.org.au/wp-content/uploads/2023/08/ASHM-Website_CDNA_xdrgonorrhoea_recommendations.pdf. Accessed 22 February 2024.

[14] Australasian Society for HIV Viral Hepatitis and Sexual Health Medicine . Australian STI management guidelines for use in primary care—gonorrhoea. https://sti.guidelines.org.au/sexually-transmissible-infections/gonorrhoea/. Accessed 24 May 2024.

[15] Clinical and Laboratory Standards Institute . Performance standards for antimicrobial susceptibility testing. 30th ed. CLSI supplement M100. Wayne, PA: Clinical and Laboratory Standards Institute, 2020.

[16] Zhu X , Xi Y, Gong X, Chen S. Ceftriaxone-resistant gonorrhea—China, 2022. MMWR Morb Mortal Wkly Rep 2024; 73:255–9. 38547027 10.15585/mmwr.mm7312a2 PMC10986818

[17] Aung ET , Chow EP, Fairley CK, et al International travel as risk factor for *Chlamydia trachomatis* infections among young heterosexuals attending a sexual health clinic in Melbourne, Australia, 2007 to 2017. Euro Surveill 2019; 24:1900219. 31690365 10.2807/1560-7917.ES.2019.24.44.1900219 PMC6836681

[18] Williamson DA , Chow EPF, Gorrie CL, et al Bridging of *Neisseria gonorrhoeae* lineages across sexual networks in the HIV pre-exposure prophylaxis era. Nat Commun 2019; 10:3988. 31488838 10.1038/s41467-019-12053-4 PMC6728426

[19] Chow EPF , Hocking JS, Ong JJ, Phillips TR, Fairley CK. Sexually transmitted infection diagnoses and access to a sexual health service before and after the national lockdown for COVID-19 in Melbourne, Australia. Open Forum Infect Dis 2021; 8:ofaa536. 33506064 10.1093/ofid/ofaa536 PMC7665697

